# Therapeutic Evaluation and Management Strategy of Transarterial Embolization for Giant Liver Hemangiomas Exceeding 10 cm in Diameter

**DOI:** 10.1007/s00270-021-02897-z

**Published:** 2021-08-18

**Authors:** Xin Li, Feng-Yong Liu, Hong-Jun Yuan, Xiao-Mei Tian, Jing Tang, Ting Ye, Kan Ji

**Affiliations:** 1 Department of Interventional Radiology, The First Medical Center of Chinese, PLA General Hospital, Beijing, 100853 P.R. China; 2 Department of Interventional Therapy, The Fifth Medical Center of Chinese, PLA General Hospital, Beijing, 100039 P.R. China

## Letter to Editor,

Transarterial embolization (TAE) is increasingly considered as the effective local treatment for hepatic hemangioma HH, having advantages as a minimal invasion, fast postoperative recovery and a low incidence of complications [1–3]. However, there are barely sufficient reports applying TAE to the treatment of giant liver hemangiomas (GLH) with a diameter of more than 10 cm. we evaluate the effectiveness of TAE in the treatment of GLH.

In this study, 25 patients with GLH receiving TAE therapy from March 2016 to June 2019 were retrospectively analyzed. The indications for GLH treatment include symptoms such as abdominal pain (4/25), a rapid increase in lesion diameter (12/25) and other related complications, such as bloating and abdominal discomfort (9/25) [4].A bleomycin-lipiodol solution (15 mg bleomycin in 5 mL of saline and 10 mL of lipiodol were mixed in a 1:2 ratio) was used to supra-selectively embolize the blood supply arteries of GLH, with gelatin sponge particles (1–2 mm in diameter) used for auxiliary embolization. SPSS 22.0 software was used for statistical analysis of data to evaluate therapy effectiveness. The data were expressed as means ± SD deviation and compared by non-parametric test (Wilcoxon signed-rank test); *P* < 0.05 was considered statistically significant.

**Fig. 1:**
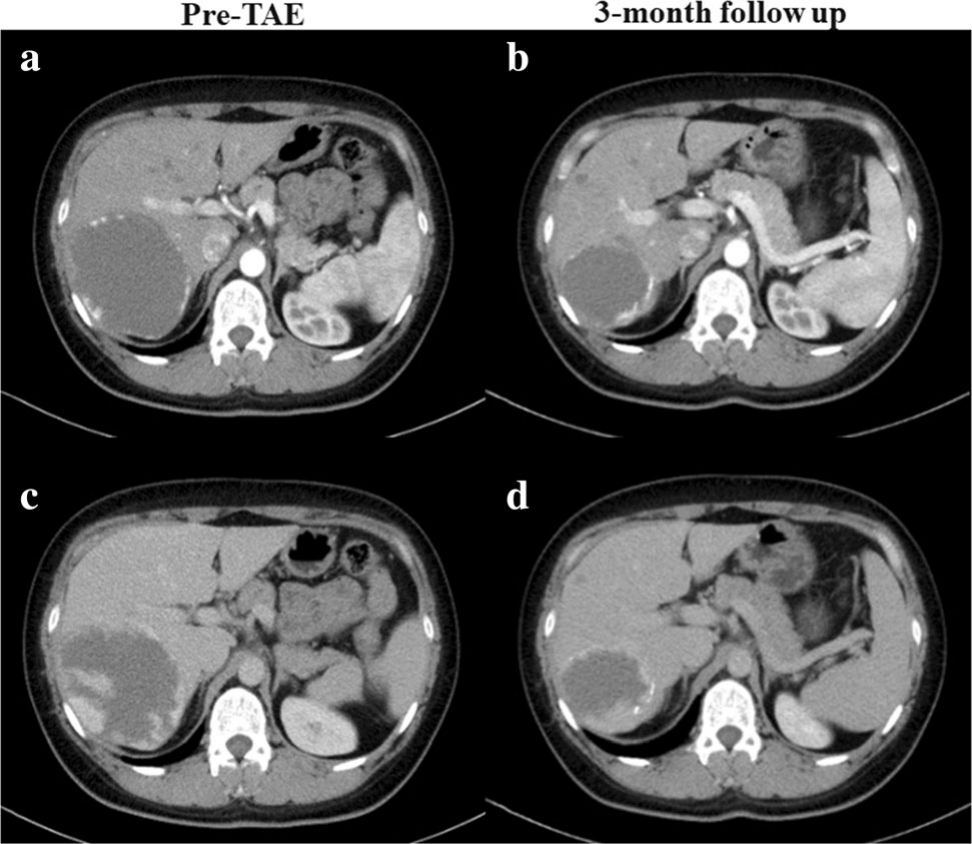
A 39-year-old woman with a 11 cm HH. CT in the arterial phase **A** and venous phase **C** showed a large hepatic hemangioma in the right liver. Three months after TAE treatment, the patient received the first re-examination and CT in the arterial phase **B** and venous phase **D** revealed the volume of the hemangioma was decreased

The average follow-up time of patients was 7.88 ± 5.49 months and the first re-examination was done 2.92 ± 0.76 months later after TAE treatment (Fig. 1). Radiological examinations suggested that the average maximal lesion diameter was reduced to 10.96 ± 3.66 cm from 13.14 ± 3.27 cm before TAE (*z* = − 4.287, *P* < 0.001) and the average maximal volume decreased from 839.43 ± 722.39 cm^3^ before TAE to 509.38 ± 459.76 cm^3^ (*z* = − 4.286, *P* < 0.001). 13 patients were followed up for the second time 9.07 ± 3.67 months later after first follow-up. Follow-up radiological examinations showed that the maximal lesion diameter and volume were decreased from 9.28 ± 2.64 cm and 343.37 ± 356.68 cm^3^ to 7.20 ± 2.37 cm (*z* = − 3.18, *P* = 0.001) and 196.26 ± 231.10 cm^3^ (*z* = -3.04, *P* = 0.002). Through follow-up observations, we confirmed that the size of lesions has a trend of gradually decreasing with time.

**Fig. 2:**
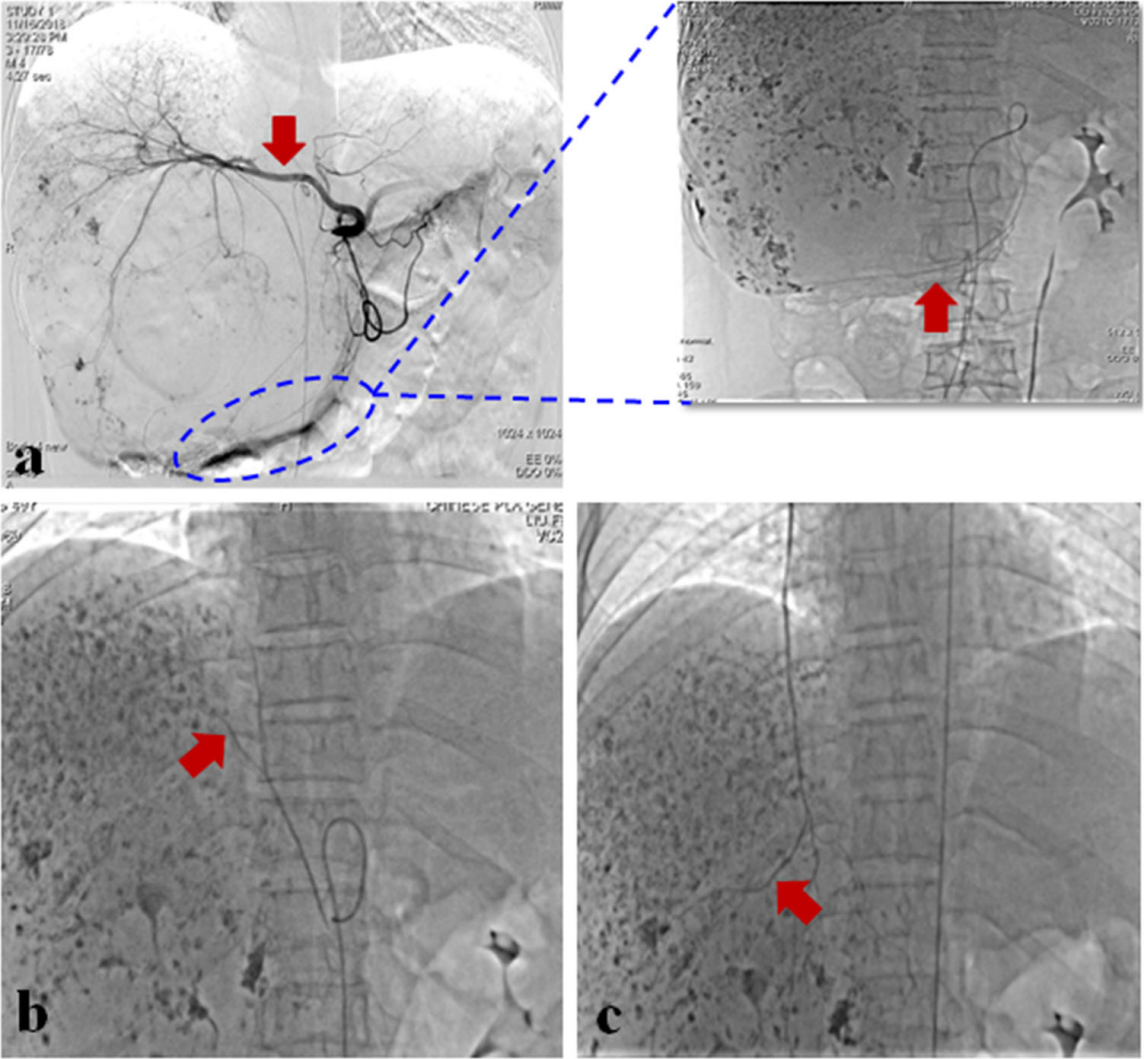
A 49-year-old woman with a 17 cm GLH in the right lobe of the liver. TAE intraoperative angiography showed that in addition to blood supply from hepatic artery **A**, the gastroduodenal artery (blue ellipse in (a)), the right inferior phrenic artery **B** and the right internal thoracic artery **C** were involved in the blood supply for GLH. The red arrows indicate the blood supply arteries

Moreover, intraoperative angiography showed that in 11 (44.00%, 11/25) patients, GLH was fed by the hepatic artery, while in the other 14 patients (56.00%, 14/25), GLH was fed both by the hepatic artery and the inferior phrenic arteries, among which 9 patients received blood supply from the right inferior phrenic artery and 5 from the bilateral inferior phrenic arteries. The internal thoracic artery (2 cases) and the gastroduodenal artery (1 case) also occasionally participated in the blood supply to GLH (Fig. 2). We believe that the main causes of changes in the blood supply to lesions include lesion size and lesion site (Table 1). When GLH lesions are in proximity to the dome of the diaphragm, they are likely to receive blood supply from the inferior phrenic arteries and internal thoracic artery, in addition to conventional blood supply from the hepatic artery. If GLH lesions are located in the porta hepatis or hepatic lobules, the likelihood of additional blood supply from left gastric artery and gastroduodenal artery increases. If GLH lesions are located in the left lobe of the liver, the left internal thoracic artery and left inferior phrenic artery are prone to involvement in the blood supply for them. Therefore, for the treatment of GLH with TAE, the arteries for blood supply should be explicitly identified to achieve the best treatment effect.

**Table 1:** Summary of the location of the lesion and blood supply

Blood Supply Location	Hepatic artery	Right inferior phrenic arteries	Bilateral inferior phrenic arteries	Right internal thoracic artery	Gastroduodenal artery
Right lobe	17	6	3	1	1
Left lobe	2	1	–	–	–
Right and left lobes	3	2	–	1	–
Right and Caudate lobe	1	–	1	–	–
Left and Caudate lobe	2	–	1	–	–
Total	25	9	5	2	1

The success rate of TAE was 100%. According to the SIR classification [5], patients developed different degrees of post-embolization syndromes such as abdominal pain, fever, transient liver dysfunction, loss of appetite, or nausea after TAE. In 21 patients, minor post-procedural complaints were observed (Class B complication). Four patients experienced serious post-embolization complaints (abdominal pain, relapsing fever, leukocytosis and liver function test abnormalities) that were considered to be Class C–D: Major therapy was not required, but complaints required hospitalization beyond 48 h. During follow-up, none of the patients were found to have serious complications related to TAE or bleomycin, such as liver failure, liver abscess, bone marrow suppression, or lung infection.

This report suggested that TAE is an effective and safe treatment method for GLH exceeding 10 cm in diameter. However, the effectiveness of TAE therapies can be ensured only when the blood supply arteries for GLH are confirmed clearly, at the same time, the follow-up time should be extended to achieve accurate evaluation over their effectiveness.
